# Target-independent high-throughput sequencing methods provide evidence that already known human viral pathogens play a main role in respiratory infections with unexplained etiology

**DOI:** 10.1080/22221751.2019.1640587

**Published:** 2019-07-23

**Authors:** Unai Pérez-Sautu, Michael Ross Wiley, María Iglesias-Caballero, Francisco Pozo, Karla Prieto, Joseph Alex Chitty, María Luz García-García, Cristina Calvo, Inmaculada Casas, Gustavo Palacios

**Affiliations:** a Influenza and Respiratory Viruses Unit, National Center for Microbiology, Instituto de Salud Carlos III (ISCIII), Madrid, Spain; b Center for Genome Sciences, United States Army Medical Research Institute of Infectious Diseases (USAMRIID), Frederick, MD, USA; c College of Public Health, University of Nebraska Medical Center, Omaha, NE, USA; d Severo Ochoa University Hospital, Madrid, Spain

**Keywords:** Pediatric respiratory infection, high-throughput sequencing, respiratory viruses, viral genomics, metagenomics

## Abstract

Despite the advanced PCR-based assays available, a fraction of the pediatric respiratory infections remain unexplained every epidemic season, and there is a perception that novel viruses might be present in these specimens. We systematically collected samples from a prospective cohort of pediatric patients with respiratory infections, that returned negative results by validated molecular RT–PCR assays, and studied them with a target-independent, high-throughput sequencing-based approach. We also included a matched cohort of children with no symptoms of respiratory infection, as a contrast study population. More than fifty percent of the specimens from the group of patients with unexplained respiratory infections were resolved. However, the higher rate of detection was not due to the presence of novel viruses, but to the identification of well-known viral respiratory pathogens. Our results show that already known viral pathogens are responsible for the majority of cases that remain unexplained after the epidemic season. High-throughput sequencing approaches that use pathogen-specific probes are easier to standardize because they ensure reproducible library enrichment and sequencing. In consequence, these techniques might be desirable from a regulatory standpoint for diagnostic laboratories seeking to benefit from the many advantages of these sequencing technologies.

## Introduction

The rate of discovery of new microbes and of new associations of microbes with health and disease has accelerated significantly in the last decade [1–4]. Many factors are contributing to this phenomenon including those that favour the true emergence of new pathogens as well as new technologies and paradigms that enable their detection and characterization [5–7]. Popular media have focused attention on biodefense and emerging infectious diseases, providing a foundation for unprecedented support of basic and translational research in host, vector, and microbe biology, as well as diagnostics and surveillance. New molecular technologies based on high-throughput sequencing (HTS) have facilitated discovery [7–10]. Appreciation that more than 75% of emerging infectious diseases represent zoonoses has also had an impact [11]. Moreover, the databases needed to recognize microbial sequences improved dramatically with the development of more sophisticated algorithms for searching and identification [12,13]. Thus, the current accepted vision is that we are in a position to tackle effectively the problem of the detection of the “unknown known” (e.g. the unexpected rare pathogen) while true reliable agnostic pathogen discovery for detection of the “unknown unknown” (e.g. a true novel pathogen) is still an art.

According to the latest Global Health Estimates from the World Health Organization, respiratory infections are among the five leading causes of death worldwide, causing near 3 million deaths in 2016 [14]. Acute respiratory infections (ARIs) account for 1–3% of deaths in children less than 5 years of age in industrialized countries and 10–25% of deaths in developing countries [14]. ARIs of viral origin are one of the main causes of hospitalization among those patients, who typically suffer more than one episode during the season [15]. Despite advances, a significant proportion of the pediatric ARIs remain without a causative agent every epidemic season [16]. This fact is not exclusive of ARIs, more than 60% of the cases of viral encephalitis, and near 65% of all deaths from gastroenteritis and from foodborne disease, remain unassigned to a specific pathogen even after syndromic laboratory testing [17–19]. Although there are multiple reasons that explain these results that are not related with diagnostic technology failure (e.g. non-infectious causes, inadequate, late or “investigational” sampling), the perception that there are pathogens lurking undetected in those specimens is widespread. Unfortunately, no comprehensive risk assessments of the threat have been performed yet. In this report, we performed a systematic study of respiratory specimens collected from a carefully characterized and highly representative, prospective cohort of pediatric cases suffering unexplained ARI, and we compared the rate of detection of pathogens by utilizing validated molecular assays, and a comprehensive sequence-independent, high-throughput sequencing-based analysis. In order to assess for the clinical relevance of the viral identifications made by HTS in the specimens collected from the unexplained cases of respiratory infections, a second cohort of age-matched healthy individuals from the same epidemiologic environment was also studied with the same methodology.

## Materials and methods

### Patients and respiratory specimens

Between September 2012 and June 2013, 1,454 children <14 years of age with a respiratory tract disease were admitted to the Severo Ochoa Hospital (Madrid, Spain), and evaluated by an attending physician. Clinical data recorded included age, gender, gestational age (when less than 37 weeks), clinical diagnosis, need and length for oxygen therapy, axillary temperature, duration of fever, total white blood cell count, C-reactive protein serum levels, presence of infiltrate/atelectasis in chest X-rays, administration of antibiotic therapy, hospital stay, and inclusion in pediatric intensive care unit. Upper respiratory tract infection (URTI) was diagnosed when rhinorrhea and/or cough were found with or without fever in the absence of wheezing, dyspnea, crackling rales, or bronchodilator use. Asthma was diagnosed on the basis of the National Asthma Education and Prevention Program guidelines [NAEPP, 2002 retrieved from https://www.nhlbi.nih.gov/files/docs/guidelines/asthmafullrpt_archive.pdf]. Acute expiratory wheezing was considered to be bronchiolitis when it occurred for the first time in children <2 years. All other episodes of acute expiratory wheezing were considered wheezing episodes. Cases were considered Pneumonia when both focal infiltrates and consolidation in chest X-rays were detected. Cases of Apparent Life-threatening Event (ALTE) were defined as an episode that is frightening to the observer and is characterized by some combination of apnea, colour change, marked change in muscle tone, choking or gagging [20]. Fever without source (FWS) was diagnosed when otherwise healthy child 3 to 24 months of age presented with fever of less than 7 days in duration and in whom alternative infectious etiologies were ruled out.

Nasopharyngeal aspirates (NPAs) were systematically taken from these patients and analysed by multiplex real-time RT–PCR based on previously published methods [21–23]. These assays allow for the detection of the main viral respiratory pathogens including: Influenza virus A, B and C, Human rhinovirus, Enterovirus, Human orthopneumovirus (Human respiratory syncytial virus), Human bocavirus, Human metapneumovirus, Adenovirus, Human rubulavirus 1–4 (Human parainfluenza virus) and Human coronavirus (229E, OC46, HKU1, and NL63). Fifty-seven of these NPAs (age range <1 month to 14 years old; 45.6% female and 54.4% male) gave negative results in all the assays and were included in the present study. The clinical presentation of these patients (referred in the text as the “Case” group) is described in Table 1.

**Table 1.** Clinical presentation of the 57 pediatric patients included in the study.

Sample	Gender	Age	Fever	Max. Temp.	Fever Length	Hypoxia	Hypoxia Length	Hosp. Length	Torax Rx	Blood Culture	WBC count	CRP test	Diagnosis	Antibiotics	ICU
1	F	27	No	NA	NA	No	NA	2	Not Performed	Not Performed	ND	ND	Bronchiolitis	No	No
2	M	4,072	No	NA	NA	Yes	3	4	No Alterations	ND	12,980	4.0	Asthma	No	No
3	M	2,717	Yes	39.0	1	Yes	2	3	No Alterations	Not Performed	ND	ND	Bronchiolitis	No	No
4	M	448	Yes	38.4	2	No	NA	3	Infiltrates/Atelectasis	ND	30,630	24.0	Pneumonia	Yes	No
5	M	1,375	No	NA	NA	Yes	1	2	Not Performed	Not Performed	ND	ND	Asthma	No	No
6	M	1,257	Yes	38.0	2	Yes	3	4	Infiltrates/Atelectasis	Negative	11,390	82.0	Asthma	No	No
7	F	24	No	NA	NA	No	NA	2	Not Performed	Not Performed	ND	ND	URTI	No	No
8	M	26	No	NA	NA	No	NA	ND	Not Performed	Not Performed	ND	ND	URTI	No	No
9	F	151	No	NA	NA	No	NA	3	No Alterations	Not Performed	6,090	2.0	Bronchiolitis	No	No
10	M	353	Yes	38.2	2	No	NA	3	No Alterations	Not Performed	ND	ND	Wheezing Episode	No	No
11	F	225	Yes	39.0	1	Yes	1	5	No Alterations	Not Performed	ND	ND	Bronchiolitis	No	No
12	F	693	Yes	38.2	3	Yes	3	ND	Infiltrates/Atelectasis	Negative	5,400	41.0	Asthma	No	No
13	M	1,275	Yes	38.9	5	Yes	7	8	Infiltrates/Atelectasis	ND	13,460	56.0	Asthma	No	No
14	F	136	Yes	38.9	2	Yes	7	9	No Alterations	Not Performed	21,150	ND	Bronchiolitis	No	No
15	M	1,245	No	NA	NA	Yes	2	3	Infiltrates/Atelectasis	Not Performed	ND	ND	Wheezing Episode	No	No
16	F	367	Yes	38.7	2	Yes	1	2	No Alterations	Not Performed	ND	ND	Bronchiolitis	No	Yes
17	F	626	Yes	38.5	1	Yes	8	8	Infiltrates/Atelectasis	ND	9,400	5.0	Bronchiolitis	No	No
18	F	511	Yes	38.5	1	Yes	2	5	Infiltrates/Atelectasis	Negative	22,480	25.0	Wheezing Episode	Yes	Yes
19	F	53	Yes	38.0	1	Yes	6	10	Infiltrates/Atelectasis	Negative	14,070	7.0	Bronchiolitis	No	No
20	F	957	No	NA	NA	Yes	6	7	No Alterations	Not Performed	ND	ND	Wheezing Episode	No	No
21	M	273	Yes	38.5	2	Yes	2	3	Not Performed	Not Performed	ND	ND	Bronchiolitis	No	Yes
22	F	1,133	Yes	38.3	1	No	NA	2	Infiltrates/Atelectasis	Negative	18,450	34.0	Wheezing Episode	Yes	No
23	M	1,590	No	NA	NA	No	NA	2	Not Performed	Not Performed	ND	ND	Wheezing Episode	No	No
24	F	969	Yes	39.5	5	Yes	1	3	Infiltrates/Atelectasis	Negative	5,900	30.0	Pneumonia	Yes	No
25	M	246	No	NA	NA	Yes	3	5	Not Performed	Not Performed	ND	ND	Wheezing Episode	No	No
26	M	690	Yes	39.6	1	Yes	3	4	No Alterations	Not Performed	ND	ND	Wheezing Episode	No	No
27	M	56	No	NA	NA	Yes	5	6	No Alterations	Not Performed	ND	ND	Bronchiolitis	No	No
28	M	188	No	NA	NA	Yes	2	3	Not Performed	Not Performed	ND	ND	Asthma	No	No
29	F	1,113	Yes	39.0	2	Yes	2	3	Infiltrates/Atelectasis	Not Performed	ND	ND	Wheezing Episode	No	No
30	F	113	No	NA	NA	Yes	1	4	Not Performed	Not Performed	ND	ND	Bronchiolitis	No	No
31	M	331	Yes	38.5	4	Yes	1	3	No Alterations	Not Performed	ND	ND	Bronchiolitis	Yes	Yes
32	M	143	Yes	39.2	3	No	NA	1	Infiltrates/Atelectasis	ND	27,980	35.0	Bronchiolitis	Yes	No
33	M	685	No	NA	NA	Yes	1	4	No Alterations	Not Performed	ND	ND	Wheezing Episode	No	No
34	F	1,013	Yes	38.8	3	Yes	1	4	Infiltrates/Atelectasis	Negative	6,920	12.0	Wheezing Episode	No	No
35	F	37	Yes	38.5	1	No	NA	9	Not Performed	Negative	15,980	8.0	FWS	Yes	No
36	M	377	Yes	39.0	3	No	NA	3	Not Performed	Negative	16,370	124.0	URTI	Yes	Yes
37	F	1,641	Yes	39.0	5	Yes	1	4	Infiltrates/Atelectasis	Negative	11,760	76.0	Pneumonia	Yes	Yes
38	F	1,702	Yes	38.0	1	Yes	1	2	Infiltrates/Atelectasis	Not Performed	ND	ND	Asthma	No	No
39	M	22	Yes	38.0	1	No	NA	2	Not Performed	Negative	10,530	1.0	URTI	No	No
40	M	1,929	No	NA	NA	Yes	1	3	No Alterations	Not Performed	17,150	28.0	Wheezing Episode	No	No
41	M	1,980	Yes	39.0	7	No	NA	2	Infiltrates/Atelectasis	Negative	9,760	7.0	Pneumonia	Yes	No
42	M	1,675	Yes	39.0	7	No	NA	5	Infiltrates/Atelectasis	Negative	6,300	27.0	Pneumonia	Yes	No
43	M	642	Yes	39.5	7	No	NA	7	No Alterations	Negative	18,470	29.0	FWS	No	No
44	F	20	No	NA	NA	ND	ND	5	Not Performed	Not Performed	9,000	0.6	ALTE	No	No
45	M	0	No	NA	NA	No	NA	2	Not Performed	ND	ND	ND	URTI	No	No
46	M	129	Yes	38.5	11	No	NA	7	Infiltrates/Atelectasis	Negative	16,200	36.0	Pneumonia	No	No
47	M	5,203	Yes	40.5	3	No	NA	3	Infiltrates/Atelectasis	ND	9,900	18.0	Asthma	Yes	No
48	M	34	No	NA	NA	No	NA	ND	Not Performed	ND	ND	ND	Bronchiolitis	No	No
49	F	1,321	Yes	38.0	1	Yes	1	2	No Alterations	ND	ND	ND	Wheezing Episode	No	No
50	M	4,205	No	NA	NA	Yes	1	2	No Alterations	Not Performed	ND	ND	Asthma	No	No
51	M	1,707	Yes	39.0	2	Yes	2	3	Infiltrates/Atelectasis	Negative	9,070	1.0	Wheezing Episode	No	No
52	F	240	Yes	38.0	2	Yes	1	3	Infiltrates/Atelectasis	Negative	12,630	75.0	Bronchiolitis	Yes	No
53	F	1,604	Yes	ND	ND	Yes	2	ND	Infiltrates/Atelectasis	Negative	21,120	ND	Asthma	No	No
54	F	2,439	No	NA	NA	Yes	1	2	Not Performed	Not Performed	ND	ND	Asthma	No	No
55	F	579	Yes	39.2	5	Yes	3	5	Infiltrates/Atelectasis	Negative	6,850	4.0	Wheezing Episode	No	No
56	F	107	Yes	38.0	2	Yes	1	3	Infiltrates/Atelectasis	Negative	41,680	50.0	Bronchiolitis	Yes	No
57	M	3,851	No	NA	NA	Yes	1	3	Infiltrates/Atelectasis	Not Performed	ND	ND	Asthma	No	No

Notes: NA: Not applicable; ND: Data not recorded (clinical analytics were performed only in those cases where a bacterial infection was suspected by the physician); Gender: M = Male, F = Female; Max. Temp.: Maximum axillary temperature expressed in Celsius; Hosp. Length = Length of hospital stay; URTI = Upper Respiratory Tract Infection; ALTE: Apparent Life-Threatening Event; FWS: Fever Without Source; WBC: White Blood Cell; CRP: C-Reactive Protein; ICU: Intensive Care Unit. Age, length of fever, length of hypoxia, and length of hospital stay are expressed in days. WBC count is expressed in cells/mm^3^. CRP levels are expressed in mg/L.

A second group of 70 NPAs taken from a prospective cohort of 21 age-matched healthy donors was included as a control (referred in the text as the “Control” group). The individuals included in the control group were children visiting the hospital for other causes (e.g. food allergy testing) with no history of respiratory infection 10 days before to 10 days after their visit. These patients came from the same geographic area as the patients with respiratory tract disease and the NPAs were collected during the same period of time covering the epidemic season of virus circulation, and analysed with the molecular assays as described above, always resulting negative.

The study was approved by the Medical Ethics Committee of the Instituto de Salud Carlos III (CEI PI 15_2012) and informed written consent was obtained from all participants.

### Virus identification by target-agnostic high-throughput sequencing analysis

All 127 respiratory specimens included in the study were processed and sequenced individually. From each NPA, a 200 μl-aliquot was homogenized by passing the sample through 1 ml sterile syringes with 25G needles (Becton Dickinson, New Jersey, USA) and by vortexing, centrifuged at 5,000×g for 10 min at room temperature, and filtered through a 0.45 μm pore-size filter (Ultrafree-MC, Millipore, Massachusetts, USA) [24,25]. Each filtrate was processed with the RNeasy Micro kit (Qiagen, Maryland, USA) performing an on-column DNA digestion following the manufacturer's protocol. RNA extracts were then amplified by SISPA RT–PCR as described previously [6,8]. Amplification products were purified with the MinElute PCR purification kit (Qiagen), sheared using the Covaris S2 instrument (Covaris, Massachusetts, USA), and used for Illumina library preparation in the Apollo 324 NGS Library Prep System with the PrepX ILM 32i DNA Library Kit (Wafergen Biosystems, California, USA). Libraries were barcoded with non-overlapping dual indexes, pooled and sequenced using either a MiSeq instrument with a MiSeq Reagent kit v2 (Illumina, California, USA) or a NextSeq 500 system with a NextSeq 500/550 Mid Output v2 kit, both with 2×151-bp reads (Supplementary Methods – Supplementary Table 2).

Data analysis was performed as follows: Cutadapt v1.7 [26], Prinseq-lite v0.20.4 [27] and Picard (http://broadinstitute.github.io/picard), were used for removal of adaptors, primers, PCR duplicates, and for quality filtering of the index (<30 Phred) and reads (<20 Phred). Removal of reads belonging to the host was performed by aligning the quality-trimmed reads to the human genome reference GRCh38 (https://www.ncbi.nlm.nih.gov/genome/guide/human) with Bowtie2 v2.1.6 [28]. After the host removal step, reads were subjected to de novo assembly using Ray v2.2 [29]. Assembled contigs (109–28,945 bp) were taxonomically identified through sequence similarity to the nucleotide collection (nr/nt) database in GenBank using NCBI BLAST v2.2.28+ (megablast and dc-megablast; *e*-value threshold of 1×10E−04). Those contigs with no match were analysed with ORFfinder (https://www.ncbi.nlm.nih.gov/orffinder/) and the identified ORFs were compared to the non-redundant protein sequences (nr) database in GenBank by BLASTx analysis (*e-value* threshold of 100). Unmapped reads were aligned to the nr database in GenBank with DIAMOND, using sensitive mode [30]. Negative controls consisting on sterile RNase-free water were processed in parallel with the respiratory specimens. Hits identified in the negative controls were considered as laboratory contaminants and not reported (Suplemmentary Methods). The results from the BLASTn and BLASTx analysis were manually curated and those contigs backed up by reads with no paired mate, as well as those that matched repeatedly to low complexity genomic regions or to endogenous and integrated viral sequences were discarded. All sequencing data generated in this study have been deposited in the NCBI SRA database under the BioProject number PRJNA528996.

### Virus detection by contig-specific RT–PCR analysis

All those NPA specimens that contained reads of any respiratory virus were analysed by RT–PCR in order to confirm the results of the HTS analysis. Primer pairs were designed with the viral nucleotide sequence information obtained in the HTS analysis (Supplementary Table 1). RT–PCR was performed in 5 µl of the same RNA extracts that were initially used in the HTS analysis. Assays were performed with the AgPath-ID One-Step RT–PCR kit (ThermoFisher Scientific, Massachusetts, USA) following the manufacturer's protocol. Amplification conditions were as follows: 30 min at 45°C, 10 min at 95°C, 40 cycles of 30 s at 95°C, 30 s at 60°C and 1 min at 72°C, followed by a final extension step of 7 min at 72°C. RT–PCR products were analysed in 2% agarose gel electrophoresis, purified with the MinElute PCR Product Purification Kit (Qiagen), and sequenced with the BigDye Terminator v3.1 Cycle Sequencing Kit (Life Technologies, California, USA).

## Results

### Viruses identified by target-agnostic high-throughput sequencing analysis

**Figure 1.**
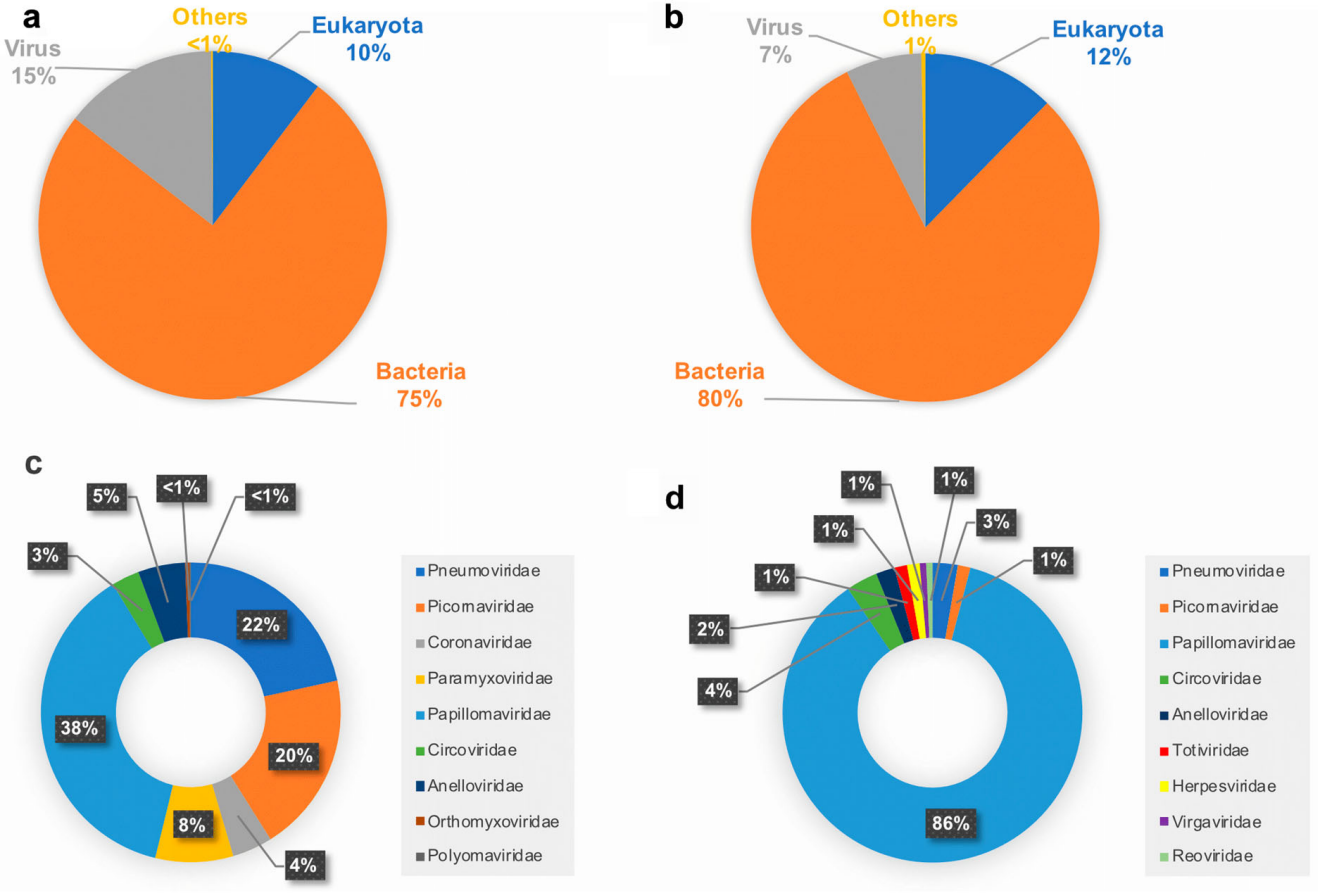
Taxonomic identification and relative abundance of the contigs from the respiratory specimens taken from the pediatric patients diagnosed with an infection of the lower respiratory tract (A and C) and of those from the specimens taken from the control group (B and D). The category “Others” includes various types of cloning, expression and mutagenesis plasmids, synthetic clones, and uncultured organism clones.

Target-agnostic HTS analysis of the 57 nasopharyngeal aspirates taken from the “Case” group produced a total of 24.6 million reads and 35,666 contigs that were taxonomically identified by BLASTn analysis (Figure 1(a)). A total of 35,226 contigs (with 42.3 million reads) were identified by BLASTn analysis from the samples taken from the “Control” group (Figure 1(b)). In the “Case” group, contigs assigned to viruses represented 14.4% (5,117 contigs), while viruses represented 7.1% (2,486 contigs) in the “Control” group (Figures 1(a,b)).

Among the 5,117 contigs assigned to viruses in the “Case” group, 13% (667 contigs with 2.6 million reads mapped) corresponded to viruses that infect eukaryotic organisms, while 87% (4,450 contigs with 4.1 million reads mapped) corresponded to bacteriophages. Among the contigs assigned to viruses that infect eukaryotic organisms 9 different families were identified namely, *Papillomaviridae*, *Pneumoviridae*, *Picornaviridae*, *Paramyxoviridae*, *Anelloviridae*, *Coronaviridae*, *Circoviridae*, *Orthomyxoviridae*, and *Polyomaviridae* (Figure 1(c)). Altogether, the number of contigs assigned to any respiratory viral pathogen accounted for 51% (340 contigs) (Table 2). When taking into account the number of reads, the vast majority (99.7%; 2.54 million reads) mapped to any of the 340 contigs assigned to respiratory viral pathogens (Table 2). Among these, Human orthopneumovirus (Human respiratory syncytial virus; HRSV) and Human rhinovirus (HRV) were the most frequently identified, followed by Enterovirus (EV) and Human rubulavirus (Human parainfluenza virus; HPIV), Human coronavirus (HCoV), Influenza B virus, and Human metapneumovirus (HMPV) (Table 2). Altogether, at least one respiratory viral pathogen could be identified by target-agnostic HTS analysis in 35 out of the 57 NPA specimens. Other viruses identified in these samples included: Sapelovirus A, Human parechovirus 1 (HPeV-1), Human papillomavirus (HPV), Human PoSCV5-like circular virus, Torque teno virus, Torque teno mini virus, and Human polyomavirus 4 (WU polyomavirus) (Table 2).

**Table 2.** Viruses that infect eukaryotic organisms identified by target-agnostic HTS in the respiratory specimens from the cases of respiratory infection and from the control group.

			Cases of respiratory infection			Control group		
Family	Genera	Virus	Contigs	Reads	Samples	Contigs	Reads	Samples
Pneumoviridae	Orthopneumovirus	Human orthopneumovirus A	120	1,669,448	14	–	–	–
		Human orthopneumovirus B	5	14,194	1	–	–	–
		Human orthopneumovirus	16	44	4	–	–	–
	Metapneumovirus	Human metapneumovirus	3	471,167	1	4	943,278	1
Picornaviridae	Enterovirus	Enterovirus B	7	739	1	–	–	–
		Enterovirus D68	11	205,365	3	–	–	–
		Rhinovirus A	27	41,472	5	2	5	1
		Rhinovirus B	13	652	2	–	–	–
		Rhinovirus C	50	22,535	8	–	–	–
		Sapelovirus A	1	115	1	–	–	–
	Parechovirus	Human parechovirus 1	21	153	1	–	–	–
Coronaviridae	Betacoronavirus	Human coronavirus HKU1	23	8,246	1	–	–	–
		Human coronavirus OC43	1	80,495	1	–	–	–
		Human coronavirus NL63	5	19	1	–	–	–
Paramyxoviridae	Rubulavirus	Human rubulavirus 4	56	29,595	4	–	–	–
Orthomyxoviridae	Betainfluenzavirus	Influenza B virus	3	18	2	–	–	–
Papillomaviridae	Betapapillomavirus	Human papillomavirus	250	4,834	37	127	6,516	24
Circoviridae	unclassified	Human PoSCV5-like circular virus	20	289	10	5	14	3
Anelloviridae	Alphatorquevirus	Torque teno virus	20	233	9	3	1,643	3
	Betatorquevirus	Torque teno mini virus	14	2,096	4	–	–	–
Polyomaviridae	Betapolyomavirus	Human polyomavirus 4 (WU polyomavirus)	1	3	1	–	–	–
Totiviridae	unclassified	Red clover powdery mildew associated totivirus	–	–	–	2	124	1
Virgaviridae	Tobamovirus	Tobacco mosaic virus	–	–	–	1	31	1
Reoviridae	Rotavirus	Rotavirus A	–	–	–	1	2	1
Herpesviridae	Cytomegalovirus	Human betaherpesvirus 5	–	–	–	2	12	1
		Total	667	2,551,712		147	951,625	

Notes: Samples were treated as two separate groups (cases of respiratory infection versus age-matched control group) and contigs and reads were grouped by virus within each group. Contigs: number of contigs that match the indicated virus. Reads: total number of reads mapping to the corresponding contigs. Samples: number of specimens where contigs and reads matching the indicated virus were identified.

From the 70 respiratory specimens taken from the “Control” group, 5.9% of the contigs (147 contigs with 951,625 reads mapped) corresponded to viruses that infect eukaryotic organisms, while 94.1% (2,339 contigs with 4.4 million reads mapped) corresponded to bacteriophages. Among the contigs assigned to viruses that infect eukaryotic organisms, members of the *Papillomaviridae*, *Circoviridae*, *Pneumoviridae*, *Anelloviridae*, *Picornaviridae*, *Herpesviridae*, *Totiviridae*, *Virgaviridae*, and *Reoviridae* were identified (Figure 1(d)). Viral respiratory pathogens were identified only in two specimens. One produced 4 contigs that were identified as HMPV, with the vast majority of the reads mapping to them (99.1%; 943,278 reads) (Table 2). In a second specimen, HRV was identified, with 2 contigs to which 5 reads mapped. The remaining 8,342 reads mapped to a total of 141 contigs identified as the following viruses: HPV, Human PoSCV5-like circular virus, Torque teno virus, Red clover powdery mildew associated totivirus, Tobacco mosaic virus, Rotavirus A, and Human betaherpesvirus 5 (Table 2).

### Viruses detected by contig-specific RT–PCR analysis

Results of the contig-specific RT–PCR assays confirmed those of the target-agnostic HTS analysis. In the “Case” group, HRSV and HRV were the most prevalent viruses, followed by EV and HPIV, HCoV, Influenza B virus, and HMPV (Table 3). In the “Control” group, the two NPA where HMPV and HRV had been identified by HTS analysis were confirmed to be positive for these viruses (Table 3).

**Table 3.** Respiratory viral pathogens identified by target-agnostic HTS analysis and confirmed by contig-specific molecular assays in the respiratory specimens from the cases of respiratory infection and from the control group.

	Cases of respiratory infection			Control group		
Virus	Contigs	Reads	Positive specimens	Contigs	Reads	Positive specimens
Human orthopneumovirus	141	1,683,686	15	–	–	–
Human metapneumovirus	3	471,167	1	4	943,278	1
Enterovirus	18	206,104	4	–	–	–
Human coronavirus	29	88,760	3	–	–	–
Rhinovirus	90	64,659	12	2	5	1
Human rubulavirus	56	29,595	4	–	–	–
Influenza B virus	3	18	2	–	–	–

Notes: Contigs: number of contigs that match the indicated virus. Reads: total number of reads mapping to the corresponding contigs. Positive specimens: number of specimens where reads matching the indicated virus were identified and confirmed by contig-specific RT-PCR analysis.

Several specimens among the “Case” group contained more than one virus (Table 4). Among these co-infections, HRSV was detected with either HPIV, EV or HRV, and HCoV was detected along with HRV (Table 4). No co-infections were detected in the “Control” group.

**Table 4.** Respiratory viral pathogens identified by target-agnostic HTS analysis and confirmed by contig-specific molecular assays in the respiratory specimens from the cases of respiratory infection.

Virus	Single infections (*n* = 29)	Coinfections (*n* = 6)	%
Human orthopneumovirus	10	–	34.5
Human metapneumovirus	1	–	3.4
Enterovirus	2	–	6.9
Human coronavirus	2	–	6.9
Rhinovirus	10	–	34.5
Human rubulavirus	2	–	6.9
Influenza B virus	2	–	6.9
Human orthopneumovirus Human rubulavirus	–	2	33.3
Human orthopneumovirus Rhinovirus	–	1	16.7
Human orthopneumovirus Enterovirus	–	2	33.3
Human coronavirus Rhinovirus	–	1	16.7

Notes: Single infections: number of specimens where only one virus was detected by contig-specific molecular assays. Coinfections: number of specimens where more than one virus was detected by contig-specific molecular assays. %: percentage of specimens positive for each virus over the total number of either single infections or coinfections.

### Specimens with unexplained results taken from cases with respiratory infection

In 22 out of the 57 (38.6%) NPAs taken from the cases of respiratory infection the target-agnostic HTS analysis did not identify any known respiratory viral pathogen (Table 5). In these samples, only bacteriophages, Torque teno virus, HPV, Human PoSCV5-like circular virus, and Human polyomavirus 4 (WU polyomavirus) were identified (Table 5).

**Table 5.** Viruses identified by target-agnostic HTS analysis in the group of respiratory specimens taken from cases of respiratory infection in which no respiratory viral pathogens were identified.

	Contigs / Reads			
Specimen	Torque teno Virus	Human PoSCV5-like circular virus	Papillomavirus	Human polyomavirus 4 (WU polyomavirus)
1	1 / 146	3 / 121	18 / 569	–
2	–	2 / 3	11 / 149	–
3	–	–	–	–
4	–	–	–	–
5	–	–	1 / 1	1 / 3
6	–	–	9 / 30	–
7	–	–	–	–
8	–	–	–	–
9	–	–	1 / 2	–
10	–	3 / 12	3 / 195	–
11	–	2 / 6	9 / 104	–
12	–	–	13 / 118	–
13	2 / 4	–	5 / 36	–
14	–	1 / 2	2 / 7	–
15	–	–	–	–
16	–	–	3 / 156	–
17	–	1 / 3	7 / 30	–
18	–	–	1 / 4	–
19	–	–	4 / 38	–
20	–	–	4 / 215	–
21	–	–	3 / 10	–
22	–	1 / 101	3 / 1,961	–

Notes: Contigs / Reads: number of contigs that match the indicated virus / total number of reads mapping to the corresponding contigs.

Further analysis of the unidentified contigs assembled from these samples by looking for sequence homologies to known viral proteins (BLASTx) returned no significative results. Although some hits to viral proteins were identified, all of these contigs mapped also to other protein sequences in the GenBank database with the same *e-values*, and with comparable ranges of coverage and identity percentage values, and thus could not be considered viral-specific hits. Representative examples of such kind of identifications are shown in Table 6. Reads that did not form contigs were mapped against the nr Genbank database with DIAMOND [30]. The analysis showed that those reads matched to proteins of bacterial or human origin or belonging to bacteriophages, and no putative novel virus was identified (results not shown).

**Table 6.** Representative examples of matches in GenBank identified by BLASTx analysis of the contigs that remained unassigned to any taxon by BLASTn analysis.

Contig	Length (bp)	Match	e-value	identity (%)	Coverage (bp)	bitscore
c42_2	372	hypothetical protein 1 [Wallerfield virus]	29	**34**.**43**	61	35
		sugar O-acyltransferase [Yokenella regensburgei]	4.8	30	60	37.0
		LysE family translocator [Pelosinus propionicus]	11	30	80	36.2
		hypothetical protein WG66_1665 [Moniliophthora roreri]	12	41.94	31	36.2
		hypothetical protein OBBRIDRAFT_890634 [Obba rivulosa]	16	41.94	31	35.8
		dynein 1 light intermediate chain [Moniliophthora roreri MCA 2997]	19	41.94	31	35.4
		hypothetical protein CONPUDRAFT_168928 [Coniophora puteana RWD-64-598 SS2]	20	41.94	31	35.4
		PREDICTED: radial spoke head 10 homolog B-like [Eufriesea mexicana]	25	42.42	33	35.0
c99_1	324	**replicase polyprotein [Porcine deltacoronavirus]**	**828**	**31**.**94**	**72**	35.4
		hypothetical protein SSIN_1445 [Streptococcus sinensis]	149	34.55	55	34.7
		hypothetical protein [Streptococcus sp. DD04]	314	34.55	55	33.9
		DNA polymerase/3’-5’ exonuclease PolX [Bacillus sp. EB01]	281	29.73	74	33.9
		hypothetical protein [Cohnella sp. 6021052837]	301	39.58	48	33.9
		hypothetical protein FisN_9Lh049 [Fistulifera solaris]	320	43.33	60	33.9
		DNA polymerase/3’-5’ exonuclease PolX [Bacillus acidiproducens]	281	29.85	67	33.1
c859_5	630	**hypothetical protein 1 [Wenzhou picorna-like virus 53]**	**1,170**	**26**.**67**	**180**	**39**.**3**
		O-methylsterigmatocystin oxidoreductase [Trametes pubescens]	377	36.11	72	39.7
		glutamine–tRNA ligase [Acinetobacter sp. WC-141]	10	25.34	221	38.5
		predicted protein [Postia placenta Mad-698-R]	12	31.36	118	38.1
		cell adhesion molecule-related/down-regulated by oncogenes [Calidris pugnax]	27	28.42	95	37.4
		hypothetical protein FOMPIDRAFT_1024183 [Fomitopsis pinicola FP-58527 SS1]	67	31.51	73	35.8
		aldehyde dehydrogenase [Thermomicrobium roseum]	72	39.29	56	35.8
		TFIIH basal transcription factor complex, subunit SSL1 [Xylona heveae TC161]	87	27.78	108	35.4
c510_1	375	**putative transposase [Saudi moumouvirus]**	**0.46**	**28**.**85**	**104**	**40**.**0**
		photosystem I reaction centre subunit XII [Nostoc sp. DB3992]	0.48	36.44	118	40.0
		photosystem I reaction centre subunit XII [Nostoc sp. KVJ20]	5.9	34.75	118	37.0
		transmembrane protein, putative [Tetrahymena thermophila SB210]	7.6	30.30	99	37.0
		phycobilisome Linker polypeptide/CpcD/allophycocyanin [Oscillatoria acuminata]	8.7	33.33	108	36.6
		CoA transferase [Candidatus Contendobacter odensis]	12	35.00	60	36.2
		1,4-alpha-glucan-branching protein [Hymenobacter gelipurpurascens]	14	31.88	69	36.2
		hypothetical protein [Enterovibrio calviensis]	14	30.11	93	35.8

We compared the bacterial hits identified by target-agnostic HTS analysis in these samples with those identified in the specimens taken from the control group. Several contigs were identified by BLASTn analysis as well-recognized bacterial respiratory pathogens, such as *Streptococcus pneumoniae*, *Haemophilus influenzae*, and *Klebsiella pneumoniae.* However, all these hits to bacterial respiratory pathogens were not exclusive of the specimens taken from the cases of respiratory infection and could be identified also in the specimens taken from the control group (results not shown). Since our analysis is not designed to be quantitative, no conclusions regarding frequency could be made.

## Discussion

Between 2012 and 2013, we tested by multiplex real-time RT–PCR [21–23] a total of 1,454 specimens from pediatric respiratory infections. Out of these, 57 remained negative after the analysis with different molecular assays. In order to shed light on the etiology of these infections, these 57 specimens were subjected to target-independent HTS analysis, along with 70 age-matched specimens from a control group. Similarly to the 57 specimens from the respiratory infections, the specimens from the control group resulted negative in the aforementioned molecular assays.

Upon HTS analysis we further identified known viral respiratory pathogens (HRSV, HRV, EV, HPIV, HCoV, HMPV and Influenza B virus). Altogether, at least one respiratory viral pathogen could be identified in 35 out of the 57 NPA specimens from the group of respiratory infection. By contrast, in the control group, only 2 samples contained contigs attributed to viral respiratory pathogens (HMPV and HRV). Moreover, the HMPV contigs detected in one of these samples, hoarded the vast majority of the reads assigned to any eukaryotic virus (99.1%). The remaining viral entities were anelloviruses and papillomaviruses common to both groups.

Identification of respiratory viruses by HTS was confirmed by contig-specific RT–PCR analysis. The HMPV and HRV identified in the control group occurred in two cases that showed no symptoms of respiratory disease. Asymptomatic infections by HRV have been previously reported and are more frequent in young children [31]. Although asymptomatic infections by HMPV can occur at the pediatric stage, they are more frequent in immunocompetent adult individuals [31–33].

Apart from common respiratory viral pathogens, Human parechovirus 1 (HPeV-1) and Human polyomavirus 4 (WU polyomavirus) were identified by HTS analysis. Both viruses were identified in specimens from the group of respiratory infection (1 sample each); none in the control group. The detection of HPeV-1 is not surprising as this virus is a frequent cause of infection in childhood, where it causes mild gastrointestinal and respiratory disease [34]. Human polyomavirus 4 (WU polyomavirus) was originally detected in respiratory secretions of a pediatric patient diagnosed with pneumonia of unknown origin, and from patients with acute respiratory co-infections [35]. Nonetheless, a subsequent larger study found no link between WU polyomavirus and acute respiratory disease [36]. In our study, WU polyomavirus was identified in one case of respiratory infection. Although no other respiratory virus was identified in this sample, WU polyomavirus presents low prevalence in cases of respiratory infection and high rates of co-infection with other common respiratory viral pathogens, and further studies are needed to ascertain its clinical significance [35–37].

Thus, out of 57, 21 (36.84%) remain unexplained from a virological standpoint, as no known respiratory or novel virus was identified. Analysis by BLASTn only returned matches to anelloviruses, papillomaviruses, and Human PoSCV5-like circular virus. In spite of thorough, in-depth analysis of those contigs with no match as well as of the unmapped reads, no viral match in the GenBank database was obtained. Anelloviruses are frequently detected in most tissues and organs, including the respiratory tract of healthy individuals, and there is no association to any disease in humans [38]. Papillomaviruses are very common worldwide, and most infections are asymptomatic and resolve spontaneously. Apart from the common warts and the various types of cancer (including an oropharyngeal form) associated to infection by HPV, low-risk types 6 and 11 are the predominant cause of respiratory papillomatosis, a disease in which noncancerous tumours grow in the air passages of the respiratory tract [39]. However, other than oropharyngeal cancer and respiratory papillomatosis, there is no evidence of an association between papillomavirus infection and respiratory disease. In addition to the lack of evidence for an association with respiratory disease, in our study anelloviruses and HPV were also identified in the control group, strengthening the conclusion that their presence was not related to the respiratory disease. Human PoSCV5-like circular virus was recently identified in respiratory secretions from an unexplained human case of febrile illness, although its association with disease was not determined [40]. Small, circular, single stranded, REP-encoding, DNA (CRESS-DNA) viruses have been increasingly identified by metagenomic HTS techniques [41] in environmental samples and in a variety of vertebrates as well as various invertebrates [42–45]. In humans, they have been reported in feces of healthy individuals [43], and in samples from unexplained cases of encephalitis and diarrhea [46], pericarditis [47], acute central nervous system infections [48], as well as in NPAs from children with respiratory infections [49]. However, neither of these studies could establish a direct association with disease. Thus, it is not clear what is the value of finding contigs that match this virus in the specimens from the group of respiratory infection. Further studies are needed to determine their role (if any) in human respiratory disease.

Our findings agree with previous studies with similar design and tools [25,50,51]. Xu et al. employed HTS to analyse a set of respiratory specimens taken from children with community-acquired pneumonia that had returned negative results in a commercial respiratory viral panel detection assay [25]. They also identified HPIV, Torque teno virus, Torque teno minivirus, and WU polyomavirus [25]. Zhou et al. studied cell-cultured supernatants with apparent cytopathic effect that had been prepared from undiagnosed respiratory specimens and identified a high prevalence of EV accompanied by HRV, HRSV, HPIV, AdV, Influenza C virus, Herpesvirus 1 and Dengue virus [51]. Taboada et al. studied nasal washings from children with respiratory infections previously found negative for common bacterial and viral respiratory pathogens by PCR, where they identified at least one known respiratory virus (including HRSV, HCoV, and HRV) in the vast majority of the specimens [50]. Neither of these studies revealed the presence of any putative novel virus on the undiagnosed respiratory infections subject of study, and the large majority of the cases could be attributed to known viral respiratory pathogens [25,50,51]. These studies suggest that all respiratory viral pathogens of clinical relevance during the pediatric stage have already been identified. In our study, we analysed 57 cases that, out of a total of 1,454, were negative in all pathogen-specific PCR assays. In consequence, only 3.9% (57/1,454) of all respiratory specimens collected during the entire epidemic season remained without a known etiology of infection after using pathogen-targeted diagnostic techniques. While target-independent HTS analysis allowed us to come to a specific diagnostic in more than half of the 57 undiagnosed infections, all the additionally resolved cases were produced by known respiratory pathogens. From these results, we can conclude that the current pathogen-specific techniques should be able to diagnose the vast majority of the respiratory infections. In consequence, we can conclude that the risk of overlooking a novel unknown viral respiratory pathogen in the pediatric population is very low when using target-specific diagnostic methods and that there is very little value in using target-independent assays. Routine virological surveillance based on target-specific techniques, such as real-time (RT)PCR or target enrichment HTS approaches (which use probes specifically designed against the viral genomic sequence of interest in order to enrich specifically for sequencing libraries derived from said virus), is appropriate and constitute a first-line diagnostic tool.

The question about the etiology behind those cases of respiratory infection that remain negative for all routine diagnostic assays have been previously addressed by several groups [24,25,50–52]. Although in our study we used an overall approach which was similar to such previous studies, we introduced several improvements to our specific study design in order to address different limitations of the previous works. The identification of viral reads in clinical specimens remains controversial because it does not necessary imply that such viruses are responsible for the symptoms observed. Many viruses can cause asymptomatic or subclinical infections, or simply be present among the normal, healthy microbiota and replicate without any pathogenic consequences. This hinders the interpretation and understanding of the results unveiled by virus discovery studies based on HTS in regard to their clinical significance [25,50,51]. In our study, we included a contrast study population (control group) formed by a prospective cohort of healthy individuals. Such healthy control group was matched at all the critical levels with the cohort of patients with unexplained infection of the respiratory tract: (i) The individuals of the control group were age-matched; (ii) They came from the same geographic area; (iii) Their samples were also taken systematically during the same time window covering the epidemic season of virus circulation. By including the control group, we were able to determine that the viruses detected in the clinical specimens taken from the patients with an infection of the respiratory tract were not circulating among the healthy population, providing evidence that such viruses were responsible for the respiratory disease observed in those cases. This would acquire special importance if a novel viral entity for which there is no previous information available is detected. In summary, the inclusion of the healthy control group allowed us to assess the clinical relevance of the viruses identified in the samples taken from the cases of unexplained respiratory infections. Whenever possible, any future viral discovery studies should include a matched healthy control group to which the viruses identified by HTS in the patients with clinical disease can be contrasted.

In addition to the inclusion of a matched healthy control group, we processed and sequenced in parallel negative controls consisting on sterile nuclease-free water (see the Materials and Methods section) and we confirmed the viral identifications made by HTS with specific RT–PCR assays. The objective of these procedures was to minimize the chances of reporting any false viral identification. It is worth highlighting also, that we performed a systematic sampling on our study groups: samples were collected from all the children arriving consecutively at the hospital during the period of the study, whose parents or legal guardians gave explicit written consent, and according to pre-established clinical and medical criteria (see the Materials and Methods section) with no further selection. By combining all the procedures discussed above, our study design constitutes a novel integrated approach that ensures a robust representativeness of the results, minimizes any possible bias, and provides a better understanding about the clinical implications of the viral identifications made by HTS analysis.

While HTS was superior in the overall rate of detection of pathogens, that was not due to the presence in the cohort of unknown pathogens, but mostly on the underperformance of molecular methods (real-time RT–PCR) against targeted pathogens. All the viruses additionally identified by HTS in our study were well-known respiratory pathogens, and no novel viruses were detected. Altogether, our results show that already known viral respiratory pathogens play a main etiologic role behind the unexplained cases of respiratory infection in the pediatric population. This is a very significant finding, because if extrapolated to other clinical syndromes and specimens, it might allow us to quantitatively assess the risk. Under that new paradigm, “Agnostic” technologies would still have a role in pathogen detection under outbreak and event situations where a true “unknown unknown” is suspected, but “Targeted” approaches would become desirable for Next-generation sequencing-based microbial diagnostic. This paradigm might be desirable from a regulatory standpoint for those diagnostic laboratories seeking to incorporate these technologies, since “Targeted” approaches allow for specific and reproducible library enrichment and thus they are easier to assess and validate.

## Supplementary Material

Supplemental Material
