# Supplementary material for: Target-independent high-throughput sequencing methods provide evidence that already known human viral pathogens play a main role in respiratory infections with unexplained etiology
Source: Emerg Microbes Infect. 2019 Jul 23;8(1):1054–65. doi: 10.1080/22221751.2019.1640587 (PMC6691886; doi:10.1080/22221751.2019.1640587)
Supplement: Supplemental Material [file TEMI_A_1640587_SM9193.zip › For_Production_Sumplemmentary_Methods_Perez_Sautu.docx]

**Target-independent high-throughput sequencing methods provide evidence that already known human viral pathogens play a main role in respiratory infections with unexplained etiology**

Unai Pérez-Sautu*^1,2^ (upsautu@gmail.com), Michael Ross Wiley^2,3^ (mike.wiley@unmc.edu), María Iglesias-Caballero^1^ (miglesias@isciii.es), Francisco Pozo^1^ (pacopozo@isciii.es), Karla Prieto^2,3^ (kprieto36@gmail.com), Joseph Alex Chitty^2^ (joseph.a.chitty2.ctr@mail.mil), María Luz García-García^4^ (lggarcia@salud.madrid.org), Cristina Calvo^4^ (ccalvorey@gmail.com), Inmaculada Casas^$1^ (icasas@isciii.es), and Gustavo Palacios^$2^ (gustavo.f.palacios.ctr@mail.mil)

^1^Influenza and Respiratory Viruses Unit, National Center for Microbiology, Instituto de Salud Carlos III (ISCIII), Majadahonda, 28220, Madrid, Spain

^2^Center for Genome Sciences, United States Army Medical Research Institute of Infectious Diseases (USAMRIID), Fort Detrick, Frederick, 21702, Maryland, USA

^3^College of Public Health, University of Nebraska Medical Center, Omaha, 68198, Nebraska, USA

^4^Severo Ochoa University Hospital, Leganés, 28911, Madrid, Spain

^$^These authors co-directed the study

*Corresponding author:

Unai Pérez-Sautu

US Army Medical Research Institute of Infectious Diseases, Center for Genome Sciences

1425 Porter St. Frederick, 21702, Maryland (USA)

Email: upsautu@gmail.com

Phone: +34 615 79 55 22

**Supplementary Materials**

**Sample processing and sequencing controls**

In order to minimize the chances of cross-contamination, carry over or read misassignment between samples, which would lead to the reporting of false viral hits, several measures were adopted. Negative controls consisting on sterile nuclease-free water (Ambion, ThermoFisher Scientific, Massachusetts, USA) were processed in parallel with the respiratory specimens and subjected to the same RNA extraction, SISPA amplification and library preparation procedures. All negative controls were individually sequenced along with the respiratory specimens, and any viral or bacterial identification made in them was considered a laboratory contamination and not reported if also present in the respiratory specimens. For library preparation and sequencing of all respiratory specimens and negative controls, a true dual-indexing configuration was used (i.e. no index was ever repeated in the libraries pooled for a given sequencing run) to ensure the maximum accuracy on demultiplexing. Finally, all viral hits were confirmed by contig-specific RT-PCR in the original RNA from the samples that was used for library preparation and sequencing.

**Additional sequencing information**

Samples were sequenced either on a MiSeq instrument with a maximum multiplexing of 16 samples per run, or in a NextSeq instrument with a maximum multiplexing of 56 samples per run. Custom, non-overlapping dual indexes in a true dual indexing configuration were used for library preparation for every sequencing run. For sequencing in the MiSeq instrument the MiSeq Reagent kit v2 kits were used. For sequencing in the NextSeq instrument the NextSeq 500/550 Mid Output v2 kits were used. All sequencing runs were performed at 2x150 bp. Detailed information about the raw read output per sample (reads with index quality score >Q30 and read quality score >Q20) is disclosed in the **Supplementary Table 2** (see below).

| **Cases of respiratory infection** | | **Control group** | |
| --- | --- | --- | --- |
| **Sample SO** | **Raw reads R1+R2** | **Sample SO** | **Raw reads R1+R2** |
| **1** | 10215520 | **58** | 53518 |
| **2** | 4167182 | **59** | 1129996 |
| **3** | 1145820 | **60** | 1504814 |
| **4** | 1104920 | **61** | 668602 |
| **5** | 1367520 | **62** | 1756970 |
| **6** | 1421328 | **63** | 2432440 |
| **7** | 2010048 | **64** | 938452 |
| **8** | 2667102 | **65** | 2482700 |
| **9** | 3022890 | **66** | 818310 |
| **10** | 1066758 | **67** | 10839824 |
| **11** | 209050 | **68** | 1059034 |
| **12** | 236346 | **69** | 1601724 |
| **13** | 56502 | **70** | 2442160 |
| **14** | 231136 | **71** | 1629138 |
| **15** | 1361248 | **72** | 1396674 |
| **16** | 1651180 | **73** | 1295916 |
| **17** | 2139146 | **74** | 930288 |
| **18** | 2980082 | **75** | 1068622 |
| **19** | 4593268 | **76** | 2317900 |
| **20** | 1085970 | **77** | 1094268 |
| **21** | 4798074 | **78** | 1257944 |
| **22** | 2427496 | **79** | 1227956 |
| **23** | 1292550 | **80** | 5256570 |
| **24** | 4356940 | **81** | 26985658 |
| **25** | 3188134 | **82** | 1101240 |
| **26** | 849606 | **83** | 2108006 |
| **27** | 654476 | **84** | 1430470 |
| **28** | 1038550 | **85** | 1829040 |
| **29** | 3560050 | **86** | 493386 |
| **30** | 2319654 | **87** | 8888306 |
| **31** | 3581966 | **88** | 1047690 |
| **32** | 3343840 | **89** | 14884080 |
| **33** | 5354032 | **90** | 1279862 |
| **34** | 1203762 | **91** | 11936956 |
| **35** | 1651974 | **92** | 2415994 |
| **36** | 1374882 | **93** | 633686 |
| **37** | 850890 | **94** | 627252 |
| **38** | 1009734 | **95** | 10410338 |
| **39** | 2570164 | **96** | 573366 |
| **40** | 1623168 | **97** | 0 |
| **41** | 3352772 | **98** | 12202796 |
| **42** | 1540252 | **99** | 0 |
| **43** | 2826156 | **100** | 636462 |
| **44** | 3771452 | **101** | 1830534 |
| **45** | 1377020 | **102** | 853264 |
| **46** | 1411738 | **103** | 769926 |
| **47** | 1399466 | **104** | 902034 |
| **48** | 2209750 | **105** | 641862 |
| **49** | 1117372 | **106** | 1637958 |
| **50** | 770800 | **107** | 13507886 |
| **51** | 1487164 | **108** | 0 |
| **52** | 1318862 | **109** | 1373276 |
| **53** | 469490 | **110** | 16171222 |
| **54** | 4541698 | **111** | 1167016 |
| **55** | 1188158 | **112** | 1521252 |
| **56** | 1523584 | **113** | 0 |
| **57** | 907768 | **114** | 5851840 |
|  |  | **115** | 2513604 |
|  |  | **116** | 1296674 |
|  |  | **117** | 591228 |
|  |  | **118** | 1642822 |
|  |  | **119** | 602174 |
|  |  | **120** | 3887404 |
|  |  | **121** | 0 |
|  |  | **122** | 2298370 |
|  |  | **123** | 0 |
|  |  | **124** | 0 |
|  |  | **125** | 666726 |
|  |  | **126** | 541546 |
|  |  | **127** | 4273238 |

**Supplementary Table 2.** Total number of raw reads produced from the group of samples taken from the cases of respiratory infection and from the samples taken from the control group.
