# Supplementary material for: Target-independent high-throughput sequencing methods provide evidence that already known human viral pathogens play a main role in respiratory infections with unexplained etiology
Source: Emerg Microbes Infect. 2019 Jul 23;8(1):1054–65. doi: 10.1080/22221751.2019.1640587 (PMC6691886; doi:10.1080/22221751.2019.1640587)
Supplement: Supplemental Material [file TEMI_A_1640587_SM9193.zip › For_Production_Supplementary_Table_1_Perez_Sautu.docx]

| **Name** | **Target** | **Sequence 5' - 3'** | **Amplicon Size** |
| --- | --- | --- | --- |
| RSVA_SP_F_1 | HRSV | CCCTCGGTGTAGTTGGAGTG | 502 bp |
| RSVA_SP_R_1 |  | TGAATGGTTGATCCGGTGGG |  |
| RSVB_F_SO10_2 |  | GCTTAGGGTTGAGATGCGGA | 553 bp |
| RSVB_R_SO10_2 |  | GTGGGCCATCTGTTGTAGGT |  |
| SO6_PIV_F1 | HPIV | CTTGAAAGCGACAGCAACCC | 318 bp |
| SO6_PIV_R1 |  | TGGGTTGTGGTGTAGAACGG |  |
| SO12_PIV_F1 |  | GGGGTAGGCTCAGGCATTTT | 743 bp |
| SO12_PIV_R1 |  | AATCTCGCAGTGGTGGTCTG |  |
| SO29_RHIN_F1 | HRV | GCTTCCCCCGTTTCACCATA | 632 bp |
| SO29_RHIN_R1 |  | CTGCCCTTCCCCAATTAGCA |  |
| SO31_RHIN_F2 |  | TGGGTGTCCGTGTTTCCTTT | 387 bp |
| SO31_RHIN_R2 |  | CCACTCCCCATAAGCAACGA |  |
| SO40_RHIN_F1 |  | GTGGTTTCCCCCGATTCACT | 232 bp |
| SO40_RHIN_R1 |  | ACTGCTCTTGGTGGTCTTGG |  |
| SO57_RHIN_F1 |  | TTTACCAACCAGGGAACGGG | 489 bp |
| SO57_RHIN_R1 |  | CATTGCCTGGTGTTTCAGCC |  |
| SO23_RHIN_F1 |  | ACATCCGCCGACCGTTTTTA | 216 bp |
| SO23_RHIN_R1 |  | CTCTGGTATGACCGCGACAA |  |
| SO29_HCoV_1F | HCoV | CCGTGGATTCTGCTCAAGGT | 520 bp |
| SO29_HCoV_1R |  | CCAATGCTATCAAGCGTGGC |  |
| SO29_HCoV_3F |  | TTGTGCAAATTACGCGGCAA | 203 bp |
| SO29_HCoV_3R |  | GAGACGGGCATCTACACTCG |  |
| SO24_EV_2F | EV | CGTGGTTGTGGGGTATGGAA | 488 bp |
| SO24_EV_2R |  | AGTTAATCCACTGGTGCGGG |  |
| SO24_EV_F |  | CGGTTACAAACAGCAAGCCG | 159 bp |
| SO24_EV_R |  | ACGAACGGGGAGGTAAACAG |  |
| SO11_Parecho_1F | HPeV | CCACAACTGTCAACACGACC | 182 bp |
| SO11_Parecho_1R |  | TCTAGCCCAGGTTGGTGTCC |  |
| SO_ADVC_2F | HMPV | TGTCATGCCAAATGGAACGC | 239 bp |
| SO_ADVC_2R |  | TTGGCTGGGTGTGGCTTATT |  |

**Supplementary Table 1**. Primer sequences and expected length of the amplification products of the contig-specific conventional RT-PCR assays derived from the sequence data information obtained from the HTS analysis.
